# The effect of *cassia fistula* emulsion on pediatric functional constipation in comparison with mineral oil: a randomized, clinical trial

**DOI:** 10.1186/2008-2231-20-83

**Published:** 2012-12-03

**Authors:** Seyyed Ali Mozaffarpur, Mohsen Naseri, Mohammad Reza Esmaeilidooki, Mohammad Kamalinejad, Ali Bijani

**Affiliations:** 1 Department of Traditional Iranian Medicine, Medical Faculty, Shahed University, Tehran, Iran; 2 Iranian Traditional Medicine Clinical trial Research Center, Shahed University, Tehran, Iran; 3 Non Communicable Pediatric Diseases Research Center, Babol University of Medical Sciences, Babol, Iran; 4 Department of Pharmacognosy, School of Pharmacy, Shahid Beheshti University of Medical Sciences, Tehran, Iran

**Keywords:** Functional constipation, Children, *Cassia fistula*, Mineral oil, Traditional Iranian Medicine (TIM), Randomized Clinical Trial (RCT)

## Abstract

**Background:**

The prevalence of Pediatric Functional Constipation (FC) has been reported between 0.7% to 29.6%. This study was conducted to compare the laxative effect of *cassia fistula* emulsion (CFE) with mineral oil (MO) on FC. Cassia fistula is named in Traditional Iranian Medicine (TIM) as “Folus”.

**Materials and methods:**

A randomized clinical trial was carried on 81 children (age range: 4–13 years) with FC, according to Rome III criteria in Amirkola Children’s Hospital, Babol, Iran. They received CFE or MO randomly for three weeks. CFE was produced according to the order of TIM references. Children were counted as improved when they exited from Rome III criteria of FC. Frequency of defecation, fecal incontinence, retentive posturing, severity of pain, consistency of stool and anal leakage of oily material were compared between the two groups and with baselines. An intent-to-treat analysis was used. Safety of drugs was assessed with the evaluation of clinical adverse effects.

**Results:**

41 children were assigned randomly to receive CFE and 40 children received MO. After three weeks of medication, 84% of children in CFE group and 50% in MO group (p = 0.002) exited from the criteria of FC, so called improved. All measurable criteria improved in both groups. The frequency of defecation in CFE group improved from 1.7 per week (before the study) to 10.6 per week (at the third week) while this parameter differed in MO group from 2 to 6.1 (p < 0.001). The severity of pain during defecation and consistency of stool improved significantly better in CFE group than MO group (p < 0.05), but there were not any significant differences between the two groups in fecal incontinence and retentive posturing. Anal leakage of oily material occurred as an important complication in MO group while the children in CFE group did not complaint it. Drug’s compliances were not significantly different in the two groups. CFE and MO did not cause clinically significant side effects.

**Conclusions:**

CFE was most effective than MO in the 3-week treatment of children with FC.

## Introduction

Functional constipation represent the common problems in childhood [1]. Constipation entails more than 3% of visits to pediatricians and 10% to 25% of visits to pediatric gastroenterologists [2,3]. The etiology is usually functional, and only a few cases are diagnosed as suffering an organic pathology [4]. Laxatives drugs mostly include bulking, osmotic, lubricating and stimulant agents [5].

Today, many people seek help from natural based drugs [6] and complementary and alternative medicine [7]. In a literature review of Traditional Iranian Medicine (TIM), we found 134 plants can be used for treatment of constipation [8]. One of the low potent and probably safe and effective plants for the treatment of constipation is *cassia fistula* (called in TIM, “Folus” or “Khiar shanbar”). The different compounds of cassia fistula are found in TIM book references [9,10].

*Cassia fistula* L. (Leguminosae) is cultivated in the tropics like the West Indies, Ceylon, China, Egypt, Amazon, Sri Lanka and many other countries, and is widely used in traditional medicine for children and pregnant women [11] as mild laxative and also as purgative [12]. Its antioxidant [13-16] and hepatoprotective effects [17-20] have been proven. Other therapeutic uses such as immunomodulator, wound healing, antifertility and antiparasitic effects in herbal medicine [11,21-26] and Ayurvedic medicine [13] have been mentioned in literatures. Any serious complication has not been reported in these studies.

Although, *cassia fistula* has cathartic and laxative effect because of the anthraquinone derivatives isolated from the pulp of the its fruits [11,12,27], we did not find any clinical trial research about it. Therefore, we designed a prospective, randomized clinical trial to compare the efficacy, safety, and patient compliance of *cassia fistula* emulsion (CFE) by a formula of TIM [10]*vs* mineral oil (MO) that is the most commonly used lubricant laxative [5,28] in the treatment of pediatric functional constipation (FC).

## Methods and materials

### Study design

This study is a clinical trial of 81 functional constipated children, referred to the Pediatric Gastroenterology Department of Amirkola Children’s Hospital (Babol, Mazandaran, IRAN), from June to September 2011.

After obtaining informed consent from the parents, the children were divided through a systematic randomization into two parallel therapeutic groups: CFE and MO.

The study was approved by the Ethics Committee of Shahed University and was registered in IRCT (ID: IRCT201107026932N1).

The patients were treated for three weeks. The investigators, the children and their parents were aware of the study group assignment.

### Inclusion criteria

Age between 4–13 years old and presence of FC were the inclusion criteria.

Functional constipation was defined by a duration of ≥2 months of at least 2 or more of the following characteristics: ≤ 2 defecation in the toilet per week, ≥ 1 episode of fecal incontinence per week, history of retentive posturing or excessive volitional stool retention, history of painful or hard bowel movements, presence of a large fecal mass in the rectum, history of large diameter stools that may obstruct the toilet. These criteria are Paris Consensus on Childhood Constipation Terminology criteria [29] as Rome III criteria of functional constipation [30].

The children with inclusion criteria were visited by the pediatric gastroenterologist to provide complete FC criteria. If history and physical examination could not prove FC, paraclinics like anorectal manometry, thyroid function tests, anti-tTG, and other laboratory tests were performed. If it was confirmed to be FC and the parents were willing the children were entered to the study.

### Exclusion criteria

Any symptom of organic constipation in the history, physical examination or paraclinics (such as hypothyroidism, Hirschsprung disease, chronic intestinal pseudo-obstruction and the presence of other chronic diseases that lead to long-term use of drugs and long-term consumption of any drug that can cause constipation.

### Drop out criteria

Intolerance of taking drugs comes in the form of nausea, vomiting, severe abdominal pain, allergic protests, incorrect use of medication and the patient's unwillingness to continue treatment

### Entering the study

All the children who were referred for treatment of FC were eligible for the study.

If FC was confirmed, the detailed history was taken and recorded in a sheet. In this sheet, the demographic information were recorded. In addition, the average of frequency defecation, retentive posturing, fecal incontinence and large bowel movement per week were questioned.

Also the parents of the children were trained to determine the average severity of pain and the average of consistency of stools defecated in the previous days without the use of any laxative. These scores were measured on the pattern of Visual Analog Scale (VAS) [31-34]. Scores for the severity of pain during defecation were ranged between 0 (without any pain) and 100 (maximum pain imaginable for parents), and also, scores for average of consistency of stools defecated were in the range between 0 (soft and comfortable) and 100 (maximum consistency imaginable for parents).

The parents learned to compare pain and consistency of stool everyday during the study with these scores. If the situation would have been better, the scores would be less than these scores, and so on.

In physical examination, the presence of an abdominal fecal mass and/or rectal mass and any anal lesion such as fissure, hemorrhoid and fistula was examined.

The treatments started with demystification. If any fecal mass was found, disimpaction was done with normal saline. Regular toilet sittings for 5 minutes after each meal and diet changes were recommended to all the children and then maintenance therapies in two groups were started.

### Safety profiles

During every visit and phone call, the parents were questioned with respect to diarrhea, abdominal pain and cramps, anal leakage, exacerbation of constipation, nausea and vomiting, dermatologic complaints, weakness, edema, palpitation or any other adverse effects.

### Follow-up

Clinical efficacy and tolerance were assessed using weekly sheets, parents completed every night. They were given three sheets (included seven questions in seven columns) to complete them daily for 3 weeks.

The parents were asked to complete the following: episodes of defecation, fecal incontinence, oily leakage and retentive posturing per day. If the defection occurred at least once a day, the average of severity of pain and consistency of stool would be written compared with what declared in the beginning of study.

In addition, the parents were asked to explain the acceptance and tolerance of drugs on the bases of our definition, from numbers 1 to 7: taking drug, with willingness, score 1. Taking with no resistance, score 2, and with objections, score 3. Taking with objection and also allurement, score 4. Taking forcefully, score 5. Does not easily take it by force, but tolerate it, score 6. Vomiting, if anyway takes it, score 7.

These concepts and scores were exactly defined and explained to the parents. In every phone call, they were asked to repeat these definitions and talked about them again to achieve their unique meaning. Once every 2–3 days we called up the parents, and if it was necessary and possible spoke with the child. In these calls, they were asked about filling out forms, therapeutic effects and any side effects of the drugs. If there were any serious questions or problems, we visited the child. In average, each child in the course of study was visited three times and had six phone calls.

At the end of 3 weeks of treatment, the children were visited by the pediatrics gastroenterologist. The filled out sheets were evaluated. If there was a serious mistake in filling out the forms, after explaining it, they were corrected by the parents.

### Preparation of CFE

The dried fruits of *cassia fistula* was purchased from local herbal drugs market in Tehran, Iran, and was identified by Herbarium of the Faculty of Pharmacy, Tehran University of Medical Sciences, where the specimens of *cassia fistula* were deposited under the voucher No.PMP-618. The dried fruits were cut into pieces and after isolating the seeds, they were soaked in sterile water. Then with a filter, the aqueous solution of *cassia fistula* was separated from the other parts. The solution was concentrated with heat and under reduced pressure and during concentration (according to TIM reference [11]) sugar and sweet almond oil was added in it, to produce heterogeneous emulsion. Every 1 ml of this emulsion contained 0.1 g of dried pulp of fruits of *cassia fistula*.

### Dosage

The children received initially either 1 ml/kg/day MO (manufactured by Hannan co., Brujen, Iran) or 0.1 g/kg body weight daily of CFE.

In Physicians’ Desk Reference for Herbal Medicines for *cassia fistula*, the daily dose of 4–8 gram of fruit pulp is recommended [35]. In TIM references, using it up to 20 gram per day is permitted [36].

To obtain an effective dose of CFE, it was used in a step-by-step increasing manner in 3 adults and then 5 children from the dose of 0.05 g/kg (from dried pulp of *cassia fistula*) in a pilot study. After this stage, it seemed that the effective safe dose of emulsion was 0.1 g/kg. So we started to prescribe it and adjust the dose every 3 days by calling up the parents. The parents were provided with guide. The results of the study lines regarding on how to adjust the dosage of medication. In case of diarrhea, the dose was reduced 25%, and if it did not respond well to medication, the dose was increased 25%, all just for one time. MO was prescribed twice daily and CFE in three-separated doses (after each meal).

### Primary outcome

The results of the study were: [1] improvement in defecation frequency per week [2] improvement in the episodes of fecal incontinence per week [3] improvement in the episodes of retentive posturing per week [4] improvement in the average of severity of pain of defecation (by VAS) [5] improvement in the average of consistency of stool defecated (by VAS) [6] patient’s drug compliance.

### Secondary outcome

Since the duration of therapy in this study was short, a prolonged follow-up was not performed thus, judging about long time recovery was not logical. However, based on criteria Rome ІІІ, all the children enrolled this study had at least two of the six criteria. To give a qualitative comparison between the two groups, the frequency of positive criteria of Rome ІІІ in children was calculated before medication, first, second and third weeks of the study to compare with one another. During 3 weeks of medication, the patients that had less than 2 of the 6 criteria were called “improved”.

### Statistical analyses

We hypothesized that CFE would be as effective or better than MO in treating FC. There was not any previous similar study. We estimated that 36 patients were required in each group to be able to detect a difference between the two groups, with 0.05 significance level at 0.80 power.

The data were entered in to SPSS (version 17), and analyzed. Efficacy analyses (improved and not improved) were performed with the intent-to-treat population, defined as all children assigned randomly. Defecation frequency, episodes of fecal incontinence, anal leakage of oil, retentive posturing, severity of pain and consistency of stool were calculated from the available follow-up data. Comparisons were made between the initial data and the follow-up data, between the first, second and third weeks follow-up data, and within the groups. The statistical analyses included the determination of means and SDs, t test, χ^2^ test, ANOVA repeated measures and Fisher’s exact test, with significance accepted at the 5% level. The results are expressed as mean ± SD or percentage.

## Result

**Figure 1:**
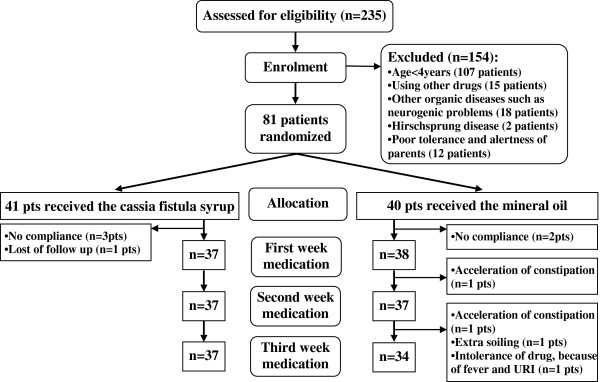
Flow chart summarizing the study process in the two treatment groups of the children with functional constipation.

From June to September 2011 among the 235 patients visited for constipation, 81 patients with FC which included 52(64.2%) boys and 29(35.8%) girls gave their written informed consent and were then randomized to receive the CFE or MO. All patients were followed-up for 3 weeks. The last follow-up visit of the patients treated in both groups was in September 2011. Figure 1 shows a flow chart revealing how the patients in both arms were selected for analysis.

The baseline characteristics of the patients in two treatment groups were similar (Table 1). The age range was between 49 to 132 months with the average of 67.7(±21.9) months and mean of 60 months. Their mean duration of constipation was 32.5(±24.3) months.

**Table 1:** **Baseline data of the children with functional constipation, in the two treatment groups of *cassia fistula *emulsion and mineral oil**

**Variable**	**CFE group (n = 41)**	**MO group (n = 40)**	**p value**
Age, months, Mean(±SD)	69.4(±24.3)	65.9(±19.1)	NS
Sex, Male, n (%)	29(70.7%)	23(57.5%)	NS
Weight, Kg(±SD)	21.7 (±7.2)	20.7(±7.8)	NS
Duration of constipation, months, Mean(±SD)	34.2(±25.9)	30.8(±22.8)	NS
Defecation ≤ 2 per week, n (%)	32(78%)	30(75%)	NS
Incontinence, n (%)	31(75.6%)	27(67.5%)	NS
History of previous treatment, n (%)	32(78%)	28(70%)	NS
Fecal impaction, n (%)	23(56.1%)	21(52.5%)	NS
Retentive posturing, n (%)	32(78%)	29(72.5%)	NS

NS: Not Significant.

In this randomized, controlled, clinical trial in children with constipation, CFE was more effective than MO for the treatment of FC.

In all our criteria, both drugs were effective. In fecal incontinence and retentive posturing, the results did not have any significant differences between the two groups. However, improvements in defecation frequency, severity of pain and consistency of stool were significantly better in CFE group.

In addition, even though oily leakage increased as a complication of MO, it did not occur in the CFE group at all. Therefore, lack of this complication is one of the most important advantages of CFE rather than MO.

In qualitative comparison between the two groups, improvement (exit from the criteria of FC) was significantly better in CFE group.

**Figure 2:**
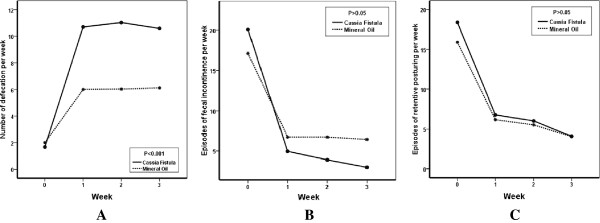
**A: Defecation frequency, B: Fecal incontinence and C: Retentive posturing (C) in the two treatment groups of *cassia fistula *emulsion and mineral oil, in the children with functional constipation, before and after medication.**

**Figure 3:**
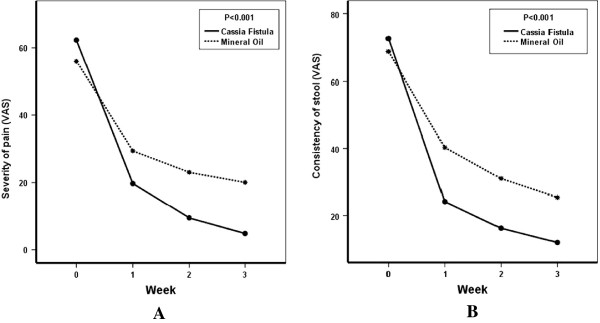
**A: Severity of pain and B: consistency of stool, in the two treatment groups of *cassia fistula *emulsion and mineral oil, in the children with functional constipation, by the score of Visual Analog Scale.**

The details of quantitative results are in Figures 2 and 3, and Table 2.

**Table 2:** **Outcome data of the children with functional constipation, in two groups of *cassia fistula *emulsion and mineral oil, before and after medication**

		**Before medication**	**First week**	**Second week**	**Third week**
Defecation /w (±SD)	*Cassia fistula*	1.7(±1.3)^NS^	10.7(±6.4)^c^	11(±6)^c^	10.6(±5.7)^c^
	Mineral oil	2(±1.7)	5.8(±4.3)	5.8(±4.3)	6.1(±4.5)
Fecal Incontinence /w (±SD)	*Cassia fistula*	19.2 (±21.7)^NS^	4.9(±10.4)^NS^	3.9(±10.1)^NS^	3(±9.1)^NS^
	Mineral oil	16.6 (±18.7)	6.1(±11.5)	6.3(±11.8)	6.4(±11.1)
Retentive Posturing/w (±SD)	*Cassia fistula*	17.7 (±19.9)^NS^	6.8(±14.5)^NS^	6(±12.5)^NS^	4.1(±8.9)^NS^
	Mineral oil	15.3 (±17.7)	5.9(±9.6)	5.5(±9.9)	4(±8.8)
severity of pain (VAS) (±SD)	*Cassia fistula*	60.9(±21.5)^NS^	19.8(±17.8)^a^	9.5(±11.7)^c^	4.8(±8.5)^c^
	Mineral oil	58.1(±22.6)	31.5(±23.1)	24.8(±21.1)	20.1(±19.9)
consistency of stool (VAS) (±SD)	*Cassia fistula*	71.9(±11.9)^NS^	24.2(±21)^c^	16.2(±16.9)^c^	11.9(±16.8)^b^
	Mineral oil	70(±15)	42.1(±22.8)	32.3(±24.2)	25.4(±22)
Acceptance and Tolerance (±SD)	*Cassia fistula*	-	2.8(±1.7)^NS^	2.5(±1.5)^NS^	2.2(±1.5)^NS^
	Mineral oil	-	2.7(±.5)	2.4(±1.4)	2.4(±1.3)

/w: per week, VAS: Visual Analog Scale, NS: Not Significant, ^a^p < 0.05 *vs* mineral oil, ^b^p < 0.01 *vs* mineral oil, ^c^p < 0.001 *vs* mineral oil.

As a qualitative comparison between the two groups, in the 1-week follow-up visit, 43% of the children in the CFE and 39% in the MO group showed improvement .These rates of the 2-week follow-up, were 62% in CFE group and 41% in MO group. After a 3-week follow-up, the improvement rate was significantly better in CFE group, so that 84% in this group exited from the criteria of functional constipation, but improvement in MO group after 3 weeks was 50% (p = 0.002). The details are in Table 3.

**Table 3:** **Comparison the effect of treatment in the children with functional constipation, in two groups of *cassia fistula *emulsion and mineral oil, before and after medication**

	**Number of present criteria/the total criteria of Rome III**	**BEFORE MEDICATION**		**FIRST WEEK**		**SECOND WEEK**		**THIRD WEEK**	
			***Cassia fistula***	**Mineral oil**	***Cassia fistula***	**Mineral oil**	***Cassia fistula***	**Mineral oil**	***Cassia fistula***	**Mineral oil**
		**(n=41)**	**(n=40)**	**(n=37)**	**(n=38)**	**(n=37)**	**(n=37)**	**(n=37)**	**(n=34)**	
Inside the criteria of Rome ІІІ	6/6	4	2	0	0	0	0	0	0
	5/6	11	9	0	4	1	2	1	3
(Not improved)	4/6	15	17	4	4	1	4	1	2
	3/6	9	6	4	7	1	8	0	5
	2/6	2	6	13	8	11	8	4	7
Out of criteria	1/6	-	-	8	8	10	9	12	8
(Improved)	0/6	-	-	8	7	13	6	19	9

As we had known, anal leakage of oily material was the most common complication of mineral oil [29]. It occurred in 27 of 38 patients (71%) with the average of 10 ± 21 times per week in the first week, in 26 of 37 patients (70%) with the average of 10.8 ± 23 times per week in the second week, and in 22 of 34 patients (64.7%) with the average of 5.5 ± 7.3 times per week in the third week. But this complication was not seen in CFE group. The most common adverse effect in CFE group was diarrhea. Twelve of the 37 patients (32%) in this group by the dose of 1 mg/kg/day had it. All of them recovered by decreasing 25% of the dose. Sputum-like stool (2 times) was seen in one patient of this group. Extra salivation in two patients and headache in one patient of MO group occurred, and recovered after the study and withdraw the drug. Abdominal pain was seen in 3 patients of each group.

In CFE group, no cases withdrew due to adverse effects.

In MO group, four patients did not complete the total duration of medication. A girl (66 months old (m/o)) withdrew after two weeks because of anal leakage of oily material for more than 20 times per day. Two patients withdrew using MO because of lack of response (A boy of 54 m/o after 2 weeks and a girl of 65 m/o after 1 week).

In addition, a girl of 62 m/o withdrew taking MO after 2 weeks because of drug intolerance due to fever and upper respiratory infection.

The average of compliance of drugs in two groups was not significantly different (Table 2). Three patients (59 m/o girl, 51 m/o girl, 71 m/o boy) in CFE group and two patients (71 m/o boy, 81 m/o girl) in MO group refused to take them, because of the medicine taste.

Only one patient (52 m/o girl) in CFE group missed to be followed-up. We mentioned her FC clinically. Nevertheless, she did not use the drug at all, and had gone to another center, underwent rectal biopsy, ruled out Hirschsprung disease and began to use poly ethylene glycol (PEG).

## Discussion

*Cassia fistula* has been used more than ten centuries in TIM [9,10,37-39]. The most important phytochemical constituents of *cassia fistula* are potent phenolic antioxidants such as anthraquinones, flavonoids and flavan-3-ol derivatives [11]. Anthraquinone is responsible for its laxative effect and so it can be categorized as stimulant laxative [5,27].

MO or liquid paraffin that was used in control group, is the most commonly used lubricant laxative [5,28]. It is composed of saturated hydrocarbons obtained from petroleum. MO acts by coating and lubricating stools, reducing colonic absorption of fecal water and facilitating the evacuation of the stools. MO is not chemically active and serious adverse effects are uncommon. Lipoid pneumonia may occur rarely because of MO aspiration [28]. We prefer to choose MO to use in control group because of its effectiveness in studies, long time use in treatment of FC with a good safety profile and fewer side effects and our health center’s long time experience on it.

Although constipation is a common chronic problem, there are very few studies with children comparing using different laxatives [40]. Consequently, there is a lack of large well designed placebo – controlled trial in this field [41].

In a comparative study by Urganci N et al., MO was more effective than lactulose in treatment of 40 children with constipation. It responded more rapidly and showed fewer side effects [42]. In another study; Hasan Karimi et al., compared MO with PEG in 103 functional constipated children. The results were better in PEG group, but no significance between the two groups [43].

Martinez-Costa C et al., used MO accompanied with senna in 62 children. Satisfactory results were achieved 1 month later in 32% of the children, 3–6 months later in 71%, and 6–12 months later in 85% [44]. Clinical trial in FC, with herbal source laxative is rare. Senna in this study was effective in 85% of children after 6–12 months accompanied with MO. but in our study, this rate of effectiveness was achieved after 3 weeks of treatment, only with CFE. Senna (*cassia angustifolia*) and *cassia fistula* are both anthranoid laxatives [27] but the prolonged use of senna leads to more prevalent and important complications than *cassia fistula*[35].

Although PEG is now one of the choices of drugs, studies are not absolute. Attar A et al., in 1999 compared a low dose PEG 3350 with lactulose. Their results were not conclusive but they said that low dose PEG 3350 was more effective than lactulose and better tolerated [45]. In 2002, Vera Loening et al., compared PEG with milk of magnesium (MOM) for 49 children. In this study, in the 12-month visit, 61% of children on PEG and 67% on MOM were doing well [46]. In another study, they compared PEG and MOM in 79 children for 12 months. The difference of efficacy was not significant between the two groups, but the acceptance of PEG was better than MOM [40].In another study PEG was found to be as effective as lactulose [47]. In 2 randomized trials, PEG with electrolytes was shown to be more effective than lactulose for 91 children over 8 weeks of therapy [48] and for 51 children over a 3-month period [49]. In another study PEG was used for 75 functional constipated children. Constipation was relieved in 85% with short-term (2 months) and in 91% with long-term (11 months) PEG therapy [3].

Although there is not any unique definition and criteria for FC, we used criteria of Rome III as inclusion criteria and for measuring qualitative outcome of the results. Our quantitative outcome measures were well defined by the use of these criteria.

For entering in the study, three steps of explanation, disimpaction and maintenance therapy were performed. Also for disimpactions, we had different choices, but we used enema with normal saline that has been effective in relieving fecal impaction [50].

Close follow-up was one of strength of this study. We called up all the parents every 3 days and talked with them about their children and in case of any changes in their bowel movement habit and other medical problems. Therefore, all of them trust us and because of this good follow-up, we missed only one patient during the study.

In this study, like more other studies in this subject, it was not possible to perform a blind study because these two drugs have different colors, tastes and smell and were administered to children in different ways. Also, because it was the first time *cassia fistula* was used in children, we should be very careful about it and its probable complications.

Another issue in this study was the age of the children entered into the study. Because we were not confident of the safety of drugs, we preferred children older than 4 years for our study. This problem led to a large number of children referred not able to complete the inclusion criteria.

On the other hand, this age of entrance caused most children entered into the study to be visited by pediatricians, before. Most patients have already received medication, but mostly without good result. Some of these patients were refractory to the treatment.

Probably, if the drug would have been used in younger children and as first choice, we could have had better responses and a lower dose of drugs might be needed.

Our baseline characteristics of patients in the two groups were well matched. They were similar to other literatures [40] except in fecal incontinence. In other studies, its frequency differs between 8 to 15.6 episodes per week [40,43,46,48,51], but in our study, it was higher to some extent. It might be because fecal impaction is one of the complications of prolonged constipation, and as our health center is a tertiary and referral one, most of the children entered in to the study had long time history of constipation and taking laxative drugs before.

A short follow-up period is a limitation of our study. One reason for this matter was that, in this study we wanted to demonstrate the effectiveness of CFE as a new drug in the treatment of FC but the Ethics Committee did not permit us to deprive the children from the usual treatment, longer than 3 weeks. This short period of follow-up is current in the first studies in other drugs in this field, for example, this period for PEG was 2 to 4 weeks [45,47]. It was 4 weeks for probiotics [52] and 2 weeks for cellulose [53]. Comparing the effectiveness and possible complications of its long-term use should be investigated in the future studies.

In our study, since the drug dose in patients with CFE was 0.1 g/kg/day, most of our patients had previous medications and might have drug resistance to some extent, so we recommend to the patients to start 0.08 to 0.1 g/ kg/day, based on dried pulp of fruits of *cassia fistula*.

There was no significant difference between the compliance of the two drugs in three weeks. The acceptance of MO in three weeks did not differ significantly, but the acceptance of CFE in the first and second week was less, but in the third week, it was better. It might be because the children found it effective and tolerated its taste.

## Conclusion

We offer CFE (*cassia fistula* emulsion) to be used for treatment of FC (pediatric functional constipation), although further researches about its safety should be done. Comparison with other laxatives such as PEG and senna for longer time is recommended.

## Abbreviations

FC: Pediatric Functional Constipation; CFE: *Cassia Fistula* Emulsion; MO: Mineral Oil; TIM: Traditional Iranian Medicine; RCT: Randomized Clinical Trial; VAS: Visual Analog Scale; PEG: Poly Ethylene Glycol.

## Competing interests

The authors have no competing interests.

## Authors’ contributions

SAM, designed and performed the research, produced the cassia fistula emulsion and wrote the paper. MN, designed the research and wrote the paper, formulated the emulsion. MRE, designed and performed the research. MK, produced the cassia fistula emulsion. AB, designed the research, analyzed the data. All authors read and approved the final manuscript.

## References

[B1] Loening-Baucke V Constipation in children N Engl J Med 1998 339 16 1155 1156 10.1056/NEJM199810153391610 9770564

[B2] Issenman RM Hewson S Pirhonen D Taylor W Tirosh A Are chronic digestive complaints the result of abnormal dietary patterns? Diet and digestive complaints in children at 22 and 40 months of age Am J Dis Child 1987 141 6 679 682 3578195 10.1001/archpedi.1987.04460060095043

[B3] Loening-Baucke V Krishna R Pashankar DS Polyethylene glycol 3350 without electrolytes for the treatment of functional constipation in infants and toddlers J Pediatr Gastroenterol Nutr 2004 39 5 536 539 10.1097/00005176-200411000-00016 15572895

[B4] Sauvat F Severe functional constipation in child: what is the solution? J Pediatr Gastroenterol Nutr 2004 38 1 10 11 10.1097/00005176-200401000-00005 14676588

[B5] Riad Rahhal AU Ronald E, Kleinman O-jG, Giorgina M-V Motility Disorder, Functional constipation Walker's Pediatric Gastrointestinal Disease fifth edition 2008 Hamilton, Ontario: Bc Decker 675 682

[B6] Salari P Nikfar S Abdollahi M A meta-analysis and systematic review on the effect of probiotics in acute diarrhea Inflamm Allergy Drug Targets 2012 11 1 3 14 22309079 10.2174/187152812798889394

[B7] van Tilburg MA Palsson OS Levy RL Feld AD Turner MJ Drossman DA Complementary and alternative medicine use and cost in functional bowel disorders: a six month prospective study in a large HMO BMC Complement Altern Med 2008 8 46 10.1186/1472-6882-8-46 18652682 PMC2499988

[B8] Mozaffarpur M Kamalinejad MR Esmaeilidooki M Yousefi M Mojahedi M Khodadust Introduction of natural materia medica, effective in the treatment of constipation, in Traditional Iranian Medicine Q J Med Hist 2012 3 9 79 95

[B9] Avecina Beirut L Al-Qanun fi al-Tibb (980–1037 A.D) al-din Is 2005 Alaalami library 289 292

[B10] Aghili SMH MM E Gharabadin Kabir(10th century AD) Vol(1) 2009 1 Tehran: Tehran University publication 936 937

[B11] Bahorun T Neergheen VS Aruoma OI Phytochemical constituents of Cassia fistula Afr J Food Agric Nutr Dev 2005 4 13 1530 1540

[B12] Iyengar MA Pendse GS Narayana N Bioassay of Cassia fistula. L. (Aragvadha) Planta Med 1966 14 3 289 301 10.1055/s-0028-1100056 5974956

[B13] Luximon-Ramma A Bahorun T Soobrattee MA Aruoma OI Antioxidant activities of phenolic, proanthocyanidin, and flavonoid components in extracts of Cassia fistula J Agric Food Chem 2002 50 18 5042 5047 10.1021/jf0201172 12188605

[B14] Manonmani G Bhavapriya V Kalpana S Govindasamy S Apparanantham T Antioxidant activity of Cassia fistula (Linn.) flowers in alloxan induced diabetic rats J Ethnopharmacol 2005 97 1 39 42 10.1016/j.jep.2004.09.051 15652272

[B15] Einstein JW Mustafa MR Nishigaki I Rajkapoor B Moh MA Protective effect of different parts of Cassia fistula on human umbilical vein endothelial cells against glycated protein-induced toxicity in vitro Methods Find Exp Clin Pharmacol 2008 30 8 599 605 10.1358/mf.2008.30.8.1268401 19088944

[B16] Bhalodia NR Nariya PB Acharya R Shukla V Evaluation of in vitro Antioxidant activity of Flowers of Cassia fistula Linn Int J PharmTech Res 2011 3 589 599

[B17] Pradeep K Raj Mohan CV Gobianand K Karthikeyan S Protective effect of Cassia fistula Linn. on diethylnitrosamine induced hepatocellular damage and oxidative stress in ethanol pretreated rats Biol Res 2010 43 1 113 125 21157638

[B18] Bhakta T Banerjee S Mandal SC Maity TK Saha BP Pal M Hepatoprotective activity of Cassia fistula leaf extract Phytomedicine 2001 8 3 220 224 10.1078/0944-7113-00029 11417916

[B19] Bhakta T Mukherjee PK Mukherjee K Banerjee S Mandal SC Maity TK Evaluation of hepatoprotective activity of Cassia fistula leaf extract J Ethnopharmacol 1999 66 3 277 282 10.1016/S0378-8741(98)00220-7 10473173

[B20] Kalantari H Jalali M Jalali A Mahdavinia M Salimi A Juhasz B Protective effect of Cassia fistula fruit extract against bromobenzene-induced liver injury in mice Hum Exp Toxicol 2011 30 8 1039 1044 10.1177/0960327110386256 20930029

[B21] Ali NH Kazmi SU Faizi S Modulation of humoral immunity by Cassia fistula and amoxy-cassia Pak J Pharm Sci 2008 21 1 21 23 18166514

[B22] Senthil Kumar M Sripriya R Vijaya Raghavan H Sehgal PK Wound healing potential of Cassia fistula on infected albino rat model J Surg Res 2006 131 2 283 289 10.1016/j.jss.2005.08.025 16242721

[B23] Yadav R Jain GC Antifertility effect of aqueous extract of seeds of Cassia fistula in female rats Adv Contracept 1999 15 4 293 301 10.1023/A:1006784224191 11145371

[B24] Gupta M Mazumder UK Rath N Mukhopadhyay DK Antitumor activity of methanolic extract of Cassia fistula L. seed against Ehrlich ascites carcinoma J Ethnopharmacol 2000 72 1–2 151 156 10967466 10.1016/s0378-8741(00)00227-0

[B25] Sartorelli P Carvalho CS Reimao JQ Ferreira MJ Tempone AG Antiparasitic activity of biochanin A, an isolated isoflavone from fruits of Cassia fistula (Leguminosae) Parasitol Res 2009 104 2 311 314 10.1007/s00436-008-1193-z 18810492

[B26] Govindarajan M Jebanesan A Pushpanathan T Larvicidal and ovicidal activity of Cassia fistula Linn. leaf extract against filarial and malarial vector mosquitoes Parasitol Res 2008 102 2 289 292 17989995 10.1007/s00436-007-0761-y

[B27] van Gorkom BA de Vries EG Karrenbeld A Kleibeuker JH Review article: anthranoid laxatives and their potential carcinogenic effects Aliment Pharmacol Ther 1999 13 4 443 452 10.1046/j.1365-2036.1999.00468.x 10215727

[B28] Bandla HP Davis SH Hopkins NE Lipoid pneumonia: a silent complication of mineral oil aspiration Pediatrics 1999 103 2 E19 10.1542/peds.103.2.e19 9925865

[B29] Benninga M Candy DC Catto-Smith AG Clayden G Loening-Baucke V Di Lorenzo C The Paris Consensus on Childhood Constipation Terminology (PACCT) Group J Pediatr Gastroenterol Nutr 2005 40 3 273 275 10.1097/01.MPG.0000158071.24327.88 15735478

[B30] Rasquin A Di Lorenzo C Forbes D Guiraldes E Hyams JS Staiano A Childhood functional gastrointestinal disorders: child/adolescent Gastroenterology 2006 130 5 1527 1537 10.1053/j.gastro.2005.08.063 16678566 PMC7104693

[B31] Philip BK Parametric statistics for evaluation of the visual analog scale Anesth Analg 1990 71 6 710 10.1213/00000539-199012000-00027 2240648

[B32] Dexter F Chestnut DH Analysis of statistical tests to compare visual analog scale measurements among groups Anesthesiology 1995 82 4 896 902 10.1097/00000542-199504000-00012 7717561

[B33] Todd KH Clinical versus statistical significance in the assessment of pain relief Ann Emerg Med 1996 27 4 439 441 10.1016/S0196-0644(96)70226-3 8604855

[B34] Kelly AM The minimum clinically significant difference in visual analogue scale pain score does not differ with severity of pain Emerg Med J 2001 18 3 205 207 10.1136/emj.18.3.205 11354213 PMC1725574

[B35] Suzanne L CBA. PDR for herbal medicine 2000 Montvale, Bergen County: Medical Economics Company Inc

[B36] Aghili SMH Shams MR Makhzan- Al' Advieh(19th century AD) 2008 Tehran: Tehran University publication 374

[B37] Abolhasan T Al-Haji HIA Elaj al-Atfal(10th century AD) 2011 Qhom: Noor e Vahy 109

[B38] Kermani NIE Institute ET Sharhe Asbab val-Alamat(15th century AD) first ed 2008 Qhom: Jalal al-Din 92 104

[B39] Jorjani SE Al-Aghraz al-Tibbia val Mabahess al-Alaiia(1042–1136 AD) University of Tehran 2006 727 729

[B40] Loening-Baucke V Pashankar DS A randomized, prospective, comparison study of polyethylene glycol 3350 without electrolytes and milk of magnesia for children with constipation and fecal incontinence Pediatrics 2006 118 2 528 535 10.1542/peds.2006-0220 16882804

[B41] van Wering HM Tabbers MM Benninga MA Are constipation drugs effective and safe to be used in children?: a review of the literature Expert Opin Drug Saf 2012 11 1 71 82 10.1517/14740338.2011.604631 21801036

[B42] Urganci N Akyildiz B Polat TB A comparative study: the efficacy of liquid paraffin and lactulose in management of chronic functional constipation Pediatr Int 2005 47 1 15 19 10.1111/j.1442-200x.2004.02001.x 15693860

[B43] Hasan Karami MK Parisa Niari Polyethylene Glycol versus Paraffin for the Treatment of Childhood Functional Constipation Iran J Pediatr 2009 19 3 255 261

[B44] Martinez-Costa C Palao Ortuno MJ Alfaro Ponce B Nunez Gomez F Martinez-Rodriguez L Ferre Franch I Functional constipation: prospective study and treatment response An Pediatr (Barc) 2005 63 5 418 425 10.1157/13080407 16266617

[B45] Attar A Lemann M Ferguson A Halphen M Boutron MC Flourie B Comparison of a low dose polyethylene glycol electrolyte solution with lactulose for treatment of chronic constipation Gut 1999 44 2 226 230 10.1136/gut.44.2.226 9895382 PMC1727381

[B46] Loening-Baucke V Polyethylene glycol without electrolytes for children with constipation and encopresis J Pediatr Gastroenterol Nutr 2002 34 4 372 377 10.1097/00005176-200204000-00011 11930092

[B47] Gremse DA Hixon J Crutchfield A Comparison of polyethylene glycol 3350 and lactulose for treatment of chronic constipation in children Clin Pediatr (Phila) 2002 41 4 225 229 10.1177/000992280204100405 12041718

[B48] Voskuijl W de Lorijn F Verwijs W Hogeman P Heijmans J Makel W PEG 3350 (Transipeg) versus lactulose in the treatment of childhood functional constipation: a double blind, randomised, controlled, multicentre trial Gut 2004 53 11 1590 1594 10.1136/gut.2004.043620 15479678 PMC1774276

[B49] Dupont C Leluyer B Maamri N Morali A Joye JP Fiorini JM Double-blind randomized evaluation of clinical and biological tolerance of polyethylene glycol 4000 versus lactulose in constipated children J Pediatr Gastroenterol Nutr 2005 41 5 625 633 10.1097/01.mpg.0000181188.01887.78 16254521

[B50] Bautista Casasnovas A Arguelles Martin F Pena Quintana L Polanco Allue I Sanchez Ruiz F Varea Calderon V Guidelines for the treatment of functional constipation An Pediatr (Barc) 2011 74 1 51 e1-7. 21123124 10.1016/j.anpedi.2010.09.017

[B51] van Dijk M Bongers ME de Vries GJ Grootenhuis MA Last BF Benninga MA Behavioral therapy for childhood constipation: a randomized, controlled trial Pediatrics 2008 121 5 e1334 e1341 10.1542/peds.2007-2402 18450876

[B52] Bu LN Chang MH Ni YH Chen HL Cheng CC Lactobacillus casei rhamnosus Lcr35 in children with chronic constipation Pediatr Int 2007 49 4 485 490 10.1111/j.1442-200X.2007.02397.x 17587273

[B53] Lu JP Huang Y Zhang Y Wang XH Shao CH Clinical trial of cellulose in treatment of functional constipation in children Zhongguo Dang Dai Er Ke Za Zhi 2011 13 5 377 380 21575341

